# Synthesis of Indole Derived Protease-Activated Receptor 4 Antagonists and Characterization in Human Platelets

**DOI:** 10.1371/journal.pone.0065528

**Published:** 2013-06-11

**Authors:** Summer E. Young, Matthew T. Duvernay, Michael L. Schulte, Craig W. Lindsley, Heidi E. Hamm

**Affiliations:** 1 Department of Pharmacology, Vanderbilt University School of Medicine, Nashville, Tennessee, United States of America; 2 Department of Chemistry, Vanderbilt University School of Medicine, Nashville, Tennessee, United States of America; 3 Vanderbilt Specialized Chemistry Center, Vanderbilt University School of Medicine, Nashville, Tennessee, United States of America; Stanford University, United States of America

## Abstract

Protease activated receptor-4 (PAR4) is one of the thrombin receptors on human platelets and is a potential target for the management of thrombotic disorders. We sought to develop potent, selective, and novel PAR4 antagonists to test the role of PAR4 in thrombosis and hemostasis. Development of an expedient three-step synthetic route to access a novel series of indole-based PAR4 antagonists also necessitated the development of a platelet based high-throughput screening assay. Screening and subsequent structure activity relationship analysis yielded several selective PAR4 antagonists as well as possible new scaffolds for future antagonist development.

## Introduction

Thrombin, a key factor in coagulation and inflammation, typically elicits cellular responses via activation of protease activated receptors (PARs). The PAR family consists of four GPCRs that are uniquely activated by proteolytic cleavage of the *N*-terminus resulting in the generation of a new *N*-terminus serving as a tethered ligand, the endogenous agonist for the receptor [1]. Human platelets express both PAR1 and PAR4. PAR1 contains a hirudin-like domain that is able to bind thrombin at sub-nanomolar concentrations however PAR4 lacks this binding domain and is considered the low affinity thrombin receptor on platelets [2]–[4]. PAR activation by thrombin in the platelet is responsible for a number of responses essential to hemostasis. Specifically, PAR-mediated platelet activation causes rapid intracellular calcium mobilization, secretion of autocrine hormones such as 5-HT and ADP, the production of eicosanoids such as thromboxane, expression of glycoproteins such as P-selectin important for platelet interaction with hematopoetic cells involved in coagulation and inflammation, and conversion of the fibrin receptor GPIIbIIIa to a high affinity conformation. PARs are attractive targets for antiplatelet and antithrombotic therapy, as both genetic and pharmacological inhibition results in blockade of arterial thrombosis in animal models [5], [6]. To date many PAR1 antagonists have been synthesized and characterized [7]–[10] and the high affinity non-peptide PAR1 antagonist, Vorapaxar, completed phase III clinical trials. The Vorapaxar TRA-CER trial was terminated due to an association of treatment with an increased risk of bleeding including intracranial hemorrhage however, the TRA-2P-TIMI 50 trial, excluding patients presenting with previous stroke, was completed and showed that PAR1 antagonism is effective in reducing cardiovascular death and ischemic events [11]–[13]. PAR4 as the low affinity thrombin receptor is consequently engaged by high concentrations of thrombin and thus subject to differential temporal engagement by thrombin. Our lab and others have documented signaling differences between PAR1 and PAR4 indicating that although activated by the same endogenous agonist and purportedly couple to the same G-proteins, platelet thrombin receptor signaling is fundamentally distinct [14]–[19]. Though the physiological role of PAR1 is becoming clear, the role of PAR4 in thrombosis and hemostasis remains unknown due to a lack of a pharmacokinetically, pharmacodynamically sufficient antagonists thus the need for potent, selective PAR4 antagonists remains.

YD-3 is the sole non-peptide, selective, PAR4 receptor antagonist [20]–[24]. The utility of YD-3 as a PAR4 antagonist has been demonstrated in *ex vivo* platelet assays [20], [23] as well as an *in vivo* mouse model of angiogeneisis [25]. The published synthetic route of YD-3 is lengthy, 9 steps beginning from cyclohexanone [20]. The inactive isomer (N^2^ instead of N^1^ of indazole becomes benzylated) comprises at least 20% of the final yield prohibiting an efficacious synthesis. A primary goal was to delete the *N^2^* indazole nitrogen and replace the core with an indole or azaindole, effectively eliminating the formation of the inactive regioisomer. In parallel, we planned to survey substituted aryl/heteroaryl moieties in multiple regions of YD-3, while also exploring replacements for, and the necessity of, the ethyl ester, a potential labile moiety. In order to rapidly determine structure activity relationships for larger libraries of analogs, we also developed a high throughput purified platelet Ca^2+^ assay to measure PAR4 mediated activation of platelets. There remains improvement not only in the synthesis of YD-3 but also in the physiochemical properties of the molecule.

## Materials and Methods

### Reagents

Purified compounds were dissolved in dimethylsulfoxide (DMSO) to a stock concentration of 10 mM and stored at −20°C until used. PAR1 activating peptide (PAR1-AP, SFLLRN) and PAR4 activating peptide (PAR4-AP, GYPGKF) were purchased from GL Biochem (Shanghai, China). NUNC 384 well plate black optical bottom was from Thermo (Rochester, NY). Fluo4-AM was purchased from Invitrogen (Eugene, Oregon). Fluorescein isothiocyanate (FITC) conjugated PAC1 and photoerythrin (PE) conjugated anti-CD62P (P-selectin) antibodies were purchased from BD Biosciences (San Jose, CA).

### Ethics Statement

Human platelets were obtained from healthy volunteers in accordance with and approved by the Vanderbilt University Institutional Review Board (050182). Written informed consent was obtained from all individuals.

### Platelet Preparation

Platelets were prepared via standard washed platelet protocol as previously described [17], [19]. Briefly, blood from healthy volunteers (averaging 30±6.6 years of age and comprised of 53% males and 47% females) was drawn into syringes containing 3.2% sodium citrate. Platelet rich plasma was prepared by centrifugation in a Forma 400 ML GP centrifuge at 1100 rpm for 15 minutes. 10X acid citrate dextrose was added to platelet rich plasma and centrifuged at 2400 rpm for 10 minutes. The supernatant was aspirated and the platelet pellet was suspended in Tyrode’s buffer containing 0.1% Bovine Serum Albumin fraction V (BSA) and counted on a Beckman Z1 Coulter particle counter (Brea, CA).

### High-throughput platelet calcium assay

Washed human platelets were prepared via standard procedure and suspended in Tyrode’s buffer containing 0.1% BSA. Platelets were dye loaded for 1 hour with Fluo4-AM in calcium assay buffer (1X HBSS without calcium or magnesium, 20 mM HEPES, 2.5 mM probenecid, 1 mM EGTA, 0.1% BSA). The calcium assay buffer containing dye is mixed with platelets to yield a final concentration of 2.5 µg/mL Fluo4-AM and 1.0×10^8^ platelets/mL. 60 µL of dye loaded platelets were added to each well of a NUNC 384 well plate black optical bottom plate (Thermo, Rochester, NY). Fluorescence measurements were recorded on a Functional Drug Screening System (FDSS) 6000, Hamamatsu (Hamamatsu, Japan) at 37°C. 10 µM of each compound was added 6 minutes prior to the addition of 80 µM PAR4-AP. Compounds were injected by the FDSS and occurred simultaneously across each plate. Experiments reported were performed in triplicate, on the same plate, from the indicated number of donors. 480∶540 (ex:em) was measured each second for a total of 12 minutes. The final concentration of DMSO in the assay was 0.5%.

### Flow Cytometry

Briefly, 60 µL of washed platelets at a concentration of 1.5×10^7^ platelets/mL were added to polystyrene tubes. Anti-CD62P antibody or PAC-1 antibody were diluted (per manufacturer protocol) in Tyrode’s buffer containing 0.1% BSA. 40 µL of diluted antibody was added to the platelets and allowed to bind for 5 minutes. Platelets were pre-treated with indicated concentrations of antagonist or DMSO control for 5 minutes followed by addition of PAR1-AP or PAR4-AP for 10 minutes. Platelet activity was quenched by the addition ice cold 1.5% paraformaldehyde followed by dilution in 1X phosphate buffered saline. The final DMSO concentration was 0.5%. Platelets were stored up to 18 hours at 4°C before flow cytometric analysis. Analysis was carried out on a BD FACS Canto II (Franklin Lakes, NJ). Fluorescent intensity was determined for 100,000 events within the platelet gate. Data was collected and analyzed via FACS DiVa software.

### Aggregation

Briefly, washed human platelets were prepared to a final concentration of 2.0×10^8^ platelets/mL in Tyrode’s buffer containing 0.1% BSA. Compounds or DMSO control were added 10 minutes prior to stimulation with either PAR1-AP or PAR4-AP. The final DMSO concentration was 0.1%. Light transmittance was recorded by a Chrono-Log Model 700 Aggregometer.

### Data Analysis

#### Calcium mobilization

The difference between basal (defined as t_0_) and maximal relative fluorescence unit (RFU) values (ΔRFUs) for ex:em 480∶540 was determined for each well. The triplicate ΔRFU for DMSO treated control wells stimulated with 80 µM PAR4-AP was averaged and the average value Δ_C_ was set to 100% max response for each plate. Each well was subsequently normalized as a percent of the Δ_C_ control (value/Δ_C_×100). Normalized data was plotted in GraphPad PRISM v.5.0.

#### Flow cytometric data analysis

100% max response was determined for each individual as the DMSO treated control stimulated with either 200 µM PAR4-AP, 20 µM PAR1-AP, or 10 nM thrombin. Samples treated with antagonist were normalized as a percentage of the DMSO treated control. Normalized data was plotted in GraphPad PRISM v.5.0. Dose response curves and subsequent IC_50_ values were generated using the ‘log(inhibitor) vs. response variable slope’ parameter. Data plotted is mean±S.E.M. or mean±S.D.

#### Platelet aggregation

The percent of light transmitted as reported by the aggregometer was recorded in GraphPad PRISM v.5.0. 100% light transmission signifies full platelet aggregation.

## Results

**Figure 1:**
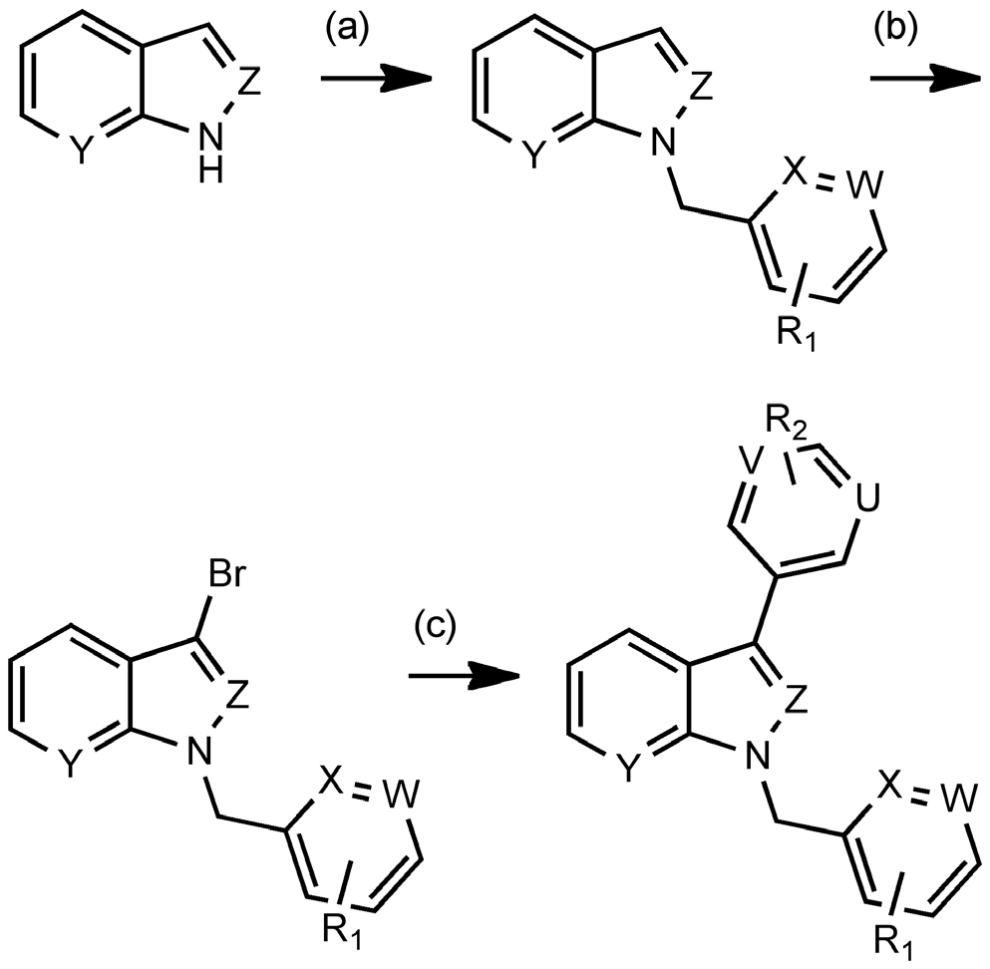
Synthetic scheme for YD-3 and novel compounds. Reagents and conditions. (a) 1.2 equiv. Ar (Het)CH_2_Br, 1.2 equiv. NaH, rt, 40 min, 39–65%; (b) 1.1 equiv. NBS, 4°C, 16h, 76–90%; (c) 10 mol% Pd_2_(dba)_3_, 20 mol% PCy_3_, 1.1 equiv. Ar(Het)B(OH)_2_ or Ar(Het)B(OR)_2_, 1.7 equiv. K_3_PO_4_, dioxane/H_2_O, mw, 120°C, 30 min, 17–56%. U,V,W,X,Y,Z = CH or N. YD-3: U,V,W,X,Y =  CH, Z = N, R_2_ = CH_2_COOCH_2_CH_3._

Although the novel synthetic route allowed for rapid generation of YD-3, the route was again subject to formation of an inactive isomer of YD-3 previously described (compound 31 in Chen et al) [20]. Initially, we planned to employ commercially available 3-bromoindoles and azaindoles, for a rapid two-step route to access analogs of YD-3, but the 3-bromo analogs proved to be unstable to the reaction conditions. Thus, we employed a multi-dimensional iterative parallel synthesis approach [26], [27] and developed an expedient three-step approach (Figure 1), beginning with indoles and azaindoles, which were *N*-alklyated with various commercially available benzyl and heteroarylmethyl bromides. A selective bromination in the 3-position [28] and a subsequent microwave-assisted Suzuki coupling [29] between brominated indoles and heteroaryl boronic acids (Figure 1) delivered YD-3 analogs (Figure S1).

**Figure 2:**
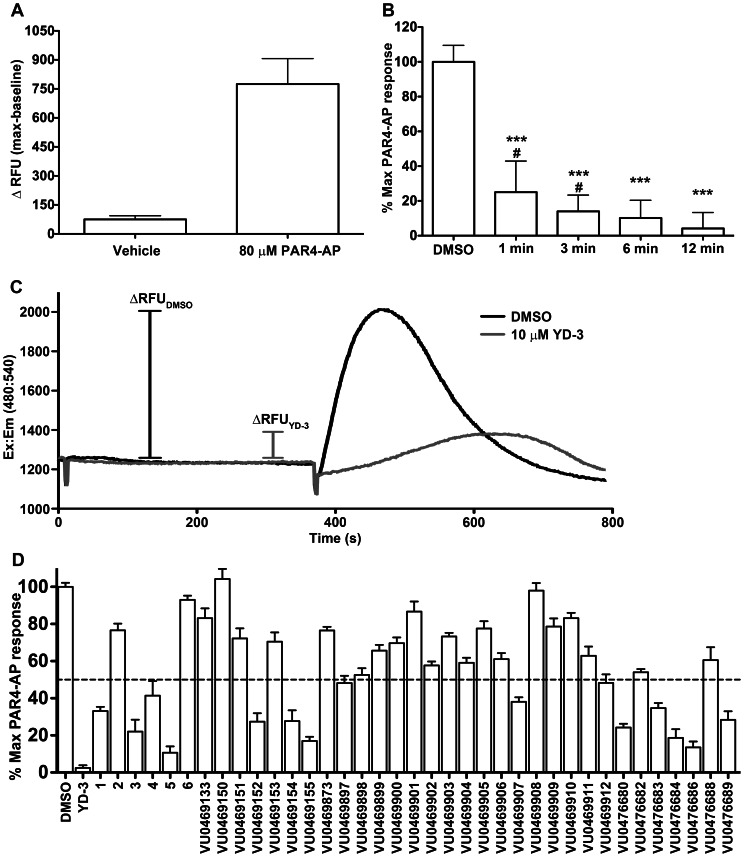
High throughput platelet calcium mobilization identifies novel indole derived PAR4 antagonists. A. To determine the Z-factor, and thus the usefulness of the assay as a screening tool, platelets were treated with either vehicle (calcium assay buffer) or 80 µM PAR4-AP in checkerboard fashion in a 384 well plate. Representative data from 1 donor is displayed as mean ± S.D. using 192 replicates of each condition in a single plate. Z-factor of the represented sample is 0.35 and a second volunteer (data not shown) was 0.31. B. Platelets were treated with 10 µM YD-3 for the indicated period of time followed by stimulation with 80 µM PAR4-AP. Data represented is an n of 2 volunteers, mean ± S.D. performed in triplicate. Means are significantly different where indicated. Unpaired one-tailed t-test: time points versus DMSO treated control ***p<0.0001. Unpaired one-tailed t-test: time points versus 12 minute time point ^#^p<0.05. C. Representative tracings of platelets treated with DMSO or 10 µM YD-3 for 6 minutes followed by stimulation with 80 µM PAR4-AP. D. Compounds were screened by treating platelets with 10 µM compound or DMSO control followed by challenge with 80 µM PAR4-AP. Data represented as percent 80 µM PAR4 response n of 2 volunteers mean±S.D. Compounds that displayed greater than 50% inhibitory activity (below dashed line) were subject to further investigation.

**Figure 3:**
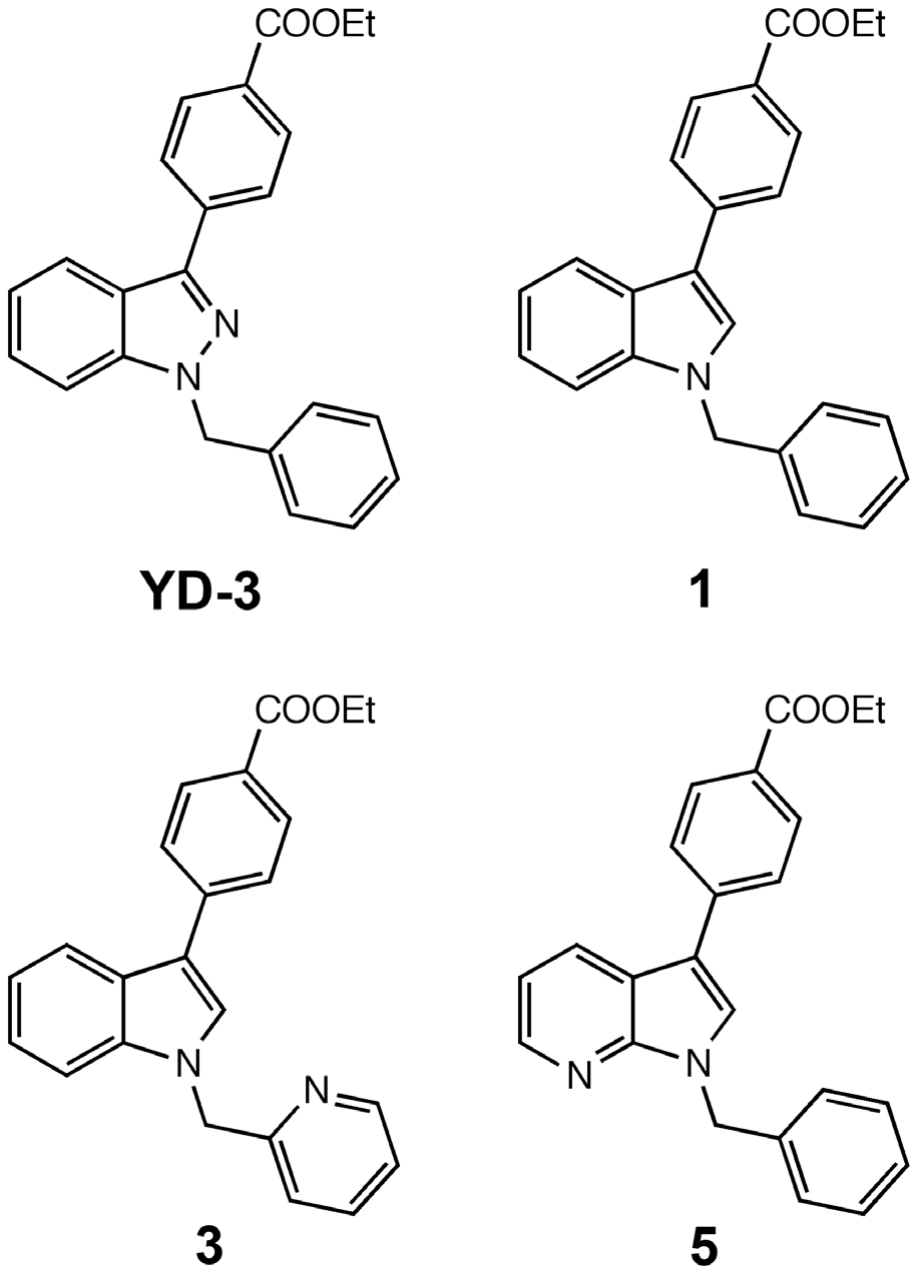
Structures of full antagonists for PAR4: YD-3, 1, 3, and 5.

**Figure 4:**
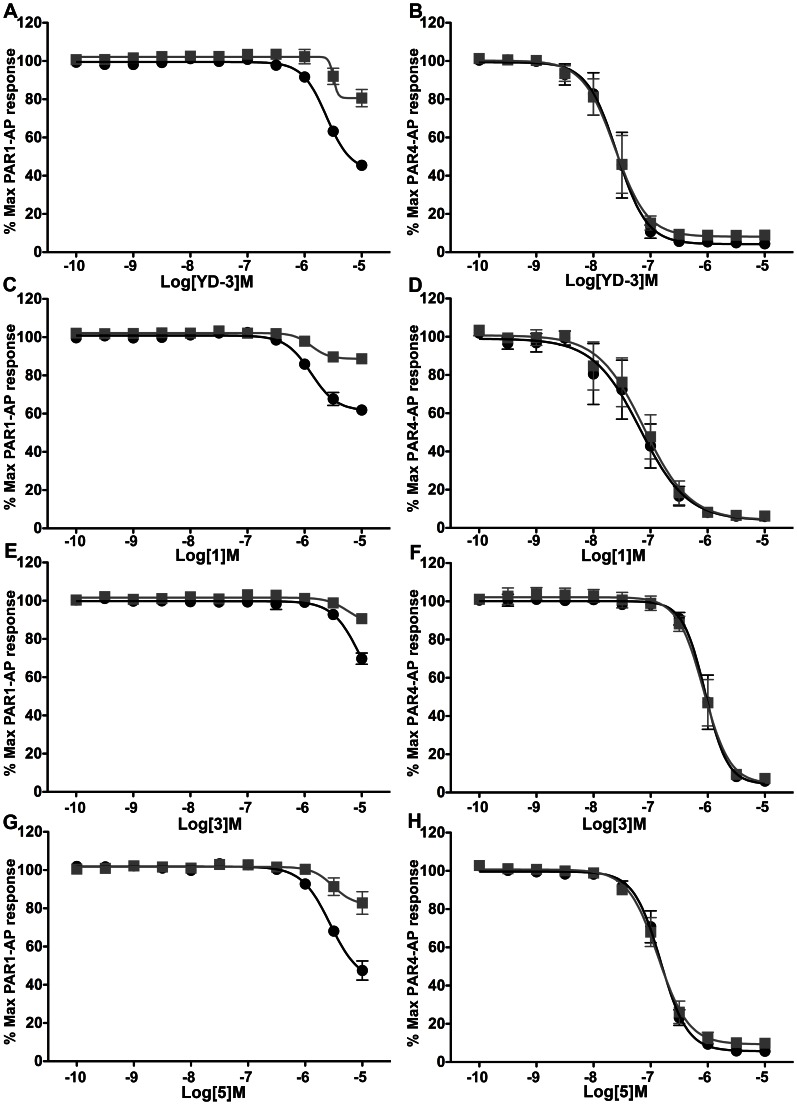
1, 3, and 5 are full PAR4 antagonists. 1, 3, and 5 were compared with YD-3 potency against PAR1 and PAR4 mediated GPIIbIIIa activation and P-selectin expression. Platelets were treated with compound or DMSO control for 5 minutes prior to stimulation with 20 µM PAR1-AP (left panels) or 200 µM PAR4-AP (right panels) for 10 minutes. GPIIbIIIa activation (black) and P-selectin expression (grey) in PAR activated platelets by flow cytometry. The order of potency for PAR4 antagonism is YD-3>1>5>3. Values are displayed as mean±S.E.M. n of 3 volunteers performed in singlicate.

Protease activated receptor-4 is coupled to Gq and stimulates intracellular calcium mobilization in platelets via the dense tubular system as well as lysosome-related acidic calcium stores [30], [31]. Measurement of intracellular calcium mobilization is a common, cost effective practice used to screen for agonists and antagonists of Gq coupled GPCRs. In our calcium assay, extracellular calcium was withheld for consistency with aggregation and flow cytometry assays, which are also carried out in calcium free buffers. The use of a low concentration of PAR4-AP made it possible to identify low affinity competitive inhibitors to better inform future structure activity relationship (SAR) studies. We validated our assay as an acceptable high throughput screening assay using the methods of Zhang et al [32]. The average Z-factor from two individuals is 0.33±0.3 (mean±S.D.) demonstrating the assay is sufficient for determining a ‘hit’ (Figure 2A). We performed an inhibition time course where samples were treated for increasing amounts of time with 10 µM YD-3 and subsequently stimulated with 80 µM PAR4-AP. We discovered significant inhibition by YD-3 occurred as early as 1 minute and longer treatment times yielded more inhibition (Figure 2B). However, 6 and 12 minutes of YD-3 treatment did not give significantly different levels of inhibition, thus we chose the 6 minute treatment time in the interest of shorter total assay time (Figure 2B). Figure 2C illustrates the kinetics of calcium mobilization for platelets stimulated with 80 µM PAR4-AP and how YD-3 can slow the mobilization of calcium as well as diminish the maximum change in fluorescence (ΔRFU). Using the high-throughput calcium mobilization assay and YD-3 as a control we determined the analogues that were effective at inhibiting a submaximal concentration of PAR4 agonist peptide (PAR4-AP) at 10 µM (Figure 2D). Similar to the SAR reported previously that led to the discovery of YD-3, SAR was very shallow, with few analogs displaying activity comparable to YD-3. From 3 rounds of synthesis we produced 38 compounds of which 1, 3, 5, VU0469152, VU046154, VU0469155, VU0469907, VU0476680, VU0476683, VU0476684, VU0476686, and VU0476689 were capable of inhibiting PAR4 mediated platelet calcium mobilization to less than 50% of the maximum response (Figure 2D), indicating that indole was a suitable replacement for the indazole core. Of note, the corresponding carboxylic acid of the terminal ethyl ester, 2, was inactive as were other ester replacements including 6, and VU0469133-912 (Figure S1). Of the compounds tested only 1, 3, and 5 were able to fully antagonize a maximal PAR4-AP response (Figure 3, Figure 4). Other ‘hits’ from the screening process did not fully antagonize PAR4 mediated GPIIbIIIa activation even at 10 µM concentration (Figure S2). The addition of chlorine in the 3 position of YD-3 was shown to give modest improvement in the potency of YD-3 for PAR4. Because of the lack of a terminal ester, we attempted to improve upon the most potent partial antagonist VU0469155, a methoxy ester derivative, by adding a chlorine in the meta position of the benzyl group (VU047689). There was a modest improvement in the potency against PAR4 but not enough to warrant further investigation (Figure S2B).

**Figure 5:**
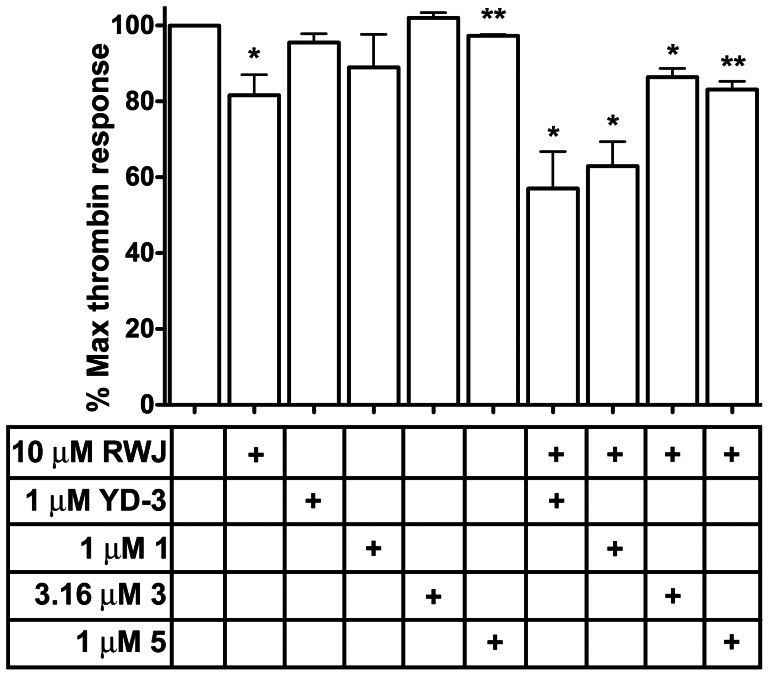
Dual PAR1/PAR4 inhibition significantly inhibits thrombin mediated platelet activation. Platelets were treated with PAR1 antagonist RWJ-56110 or PAR4 full antagonists either alone or in combination prior to stimulation with 10 nM thrombin for 10 minutes. GPIIbIIIa activation was measured, data reported is mean ± S.E.M. n of 3 volunteers. One sample t-test for significant deviation from DMSO control p-values *p<0.05, **p<0.005.

In human platelets, in addition to receptor mediated calcium mobilization via coupling to Gq, PAR stimulation converts the fibrin receptor GPIIbIIIa to its high affinity fibrin binding conformation, and triggers granule secretion containing P-selectin molecules (CD62P). The PAC-1 antibody binds only the high affinity conformation of GPIIbIIIa and is an accurate readout for GPIIbIIa activation. We measured potency of full antagonists against both PAR1 and PAR4 mediated GPIIbIIIa activation (via PAC-1 binding) and P-selectin expression (anti-CD62p binding) using flow cytometry. We did not observe any notable functional selectivity between the molecules ability to inhibit GPIIbIIIa activation (Figure 4, black) and P-selectin expression (Figure 4, grey). 1 (Figure 4D) was less potent than YD-3 (Figure 4B, Figure S4) at inhibiting PAR4 mediated responses demonstrating IC_50_ values for PAR4 mediated GPIIbIIIa activation of 66±1 nM versus 26±1 nM respectively. However, YD-3 inhibited PAR1-AP mediated GPIIbIIIa activation by 55% at 10 µM, a previously undocumented observation (Figure 4A). Interestingly, 1 reduced maximum PAR1-AP GPIIbIIIa response by 38% (Figure 4C) and 3 and 5 inhibited PAR1 by 31% (Figure 4E) 56% (Figure 4F) respectively. Together these data suggest that addition of nitrogen into the indole core may contribute to off target effects against PAR1. Both 3 and 5 were significantly less potent then either YD-3 or 1 with IC_50_’s for PAR4 mediated GPIIbIIIa activation of 1.0±1.1 µM and 170±1 nM respectively (Figure 4F, 4H). These data suggest that indole can serve as a highly selective PAR4 inhibitor with only modest loss of potency compared to YD-3.

The PAR4 partial antagonists: VU0469152, VU046154, VU0469155, VU0469907, VU0476680, VU0476683, VU0476684, VU0476686, and VU0476689, displayed a similar magnitude of inhibition against PAR1 mediated platelet activation as YD-3, 1, 3, and 5 (Figure S3) however, because these compounds did not fully antagonize PAR4 mediated platelet responses even at 10 µM, the additional loss of selectivity indicates poor lead compounds.

Inhibition of the PAR4-AP does not completely describe the potency of any PAR4 antagonist. Compounds should elicit an inhibitory effect against thrombin either alone or in combination with a PAR1 antagonist, such as RWJ-56110 [8]. Against a high dose of thrombin where both PAR1 and PAR4 are engaged [3], PAR4 antagonists alone cannot significantly inhibit thrombin mediated GPIIbIIIa activation. We subsequently tested the ability of 1 µM YD-3, 1 µM 1, 3.16 µM 3, and 1 µM 5 to inhibit thrombin mediated GPIIbIIIa activation at concentrations where each compound previously demonstrated complete inhibition of PAR4 mediated GPIIbIIIa activation and low inhibition for PAR1 mediated GPIIbIIIa activation. YD-3, 1, 3, and 5 were able to reduce PAR1 mediated GPIIbIIIa activation by 8.4±2.4%, 14.1±2.8%, 7.2±1.3%, and 7.2±1.4% respectively when used at these lower concentrations (Figure 4A, 4C, 4E, 4G). 10 µM RWJ-56110 alone was able to significantly inhibit thrombin mediated GPIIbIIIa activation by 18.4±5.4% and when used in combination with either YD-3 or 1, was able to reduce thrombin mediated signaling by 42.9±9.7% and 37.1±6.4% respectively (Figure 5). RWJ and YD-3 demonstrated dual inhibition of PAR1 and PAR4 against thrombin since the experimentally determined reduction in thrombin mediated GPIIbIIIa activation by the combination of RWJ and YD-3 (42.9±9.7%) was greater than the addition of the effect of RWJ-56110 on thrombin and the effect of YD-3 on PAR1-AP (26.8±6.0%). Compound 1 in conjunction with RWJ-56110 also slightly inhibited thrombin mediated GPIIbIIIa activation to a greater extent than addition of the inhibitory effects of each alone (37.1±6.4% compared to 32.5±6.1%). Neither 3 (14±2% reduction) nor 5 (17±2% reduction) when added in conjunction with RWJ-56110 were able to inhibit thrombin mediated platelet GPIIbIIIa activation to a greater magnitude than RWJ-56110 alone. These data suggest that even slight losses of antagonist potency against PAR4-AP responses translates into dramatic reductions in potency against thrombin. Additionally, these data suggest that for platelets stimulated with high concentrations of thrombin, the contribution of PAR1 signaling to GPIIbIIIa activation is greater than PAR4.

**Figure 6:**
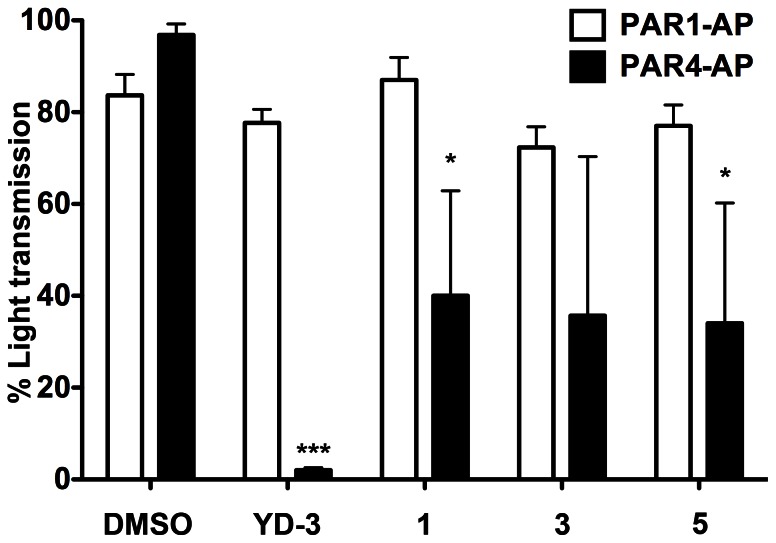
Full PAR4 antagonists inhibit PAR4 and not PAR1 mediated platelet aggregation. Platelets were treated with YD-3, 1, 3 or 5 for 10 minutes prior to stimulation with either 20 µM PAR1-AP (white bars) or 200 µM PAR4-AP (black bars). Data reported as % light transmittance where 100% represents fully aggregated platelets. Unpaired two-tailed t-test versus DMSO treated agonist control. Data displayed as mean±S.E.M n of 3 or more. Means are significantly different where indicated. ***p<0.0001, *0p<0.05.

The activities of the full antagonists YD-3, 1, 3, and 5, were assessed via classical measurement of platelet activity, platelet aggregation. We demonstrate using YD-3 as a control, 1, and 5 are able to significantly inhibit PAR4 but not PAR1 mediated platelet aggregation in healthy subjects (Figure 6). Compound 3 also inhibits PAR4 mediated platelet aggregation versus PAR1 mediated platelet aggregation though non-significantly (p = 0.054).

**Figure 7:**
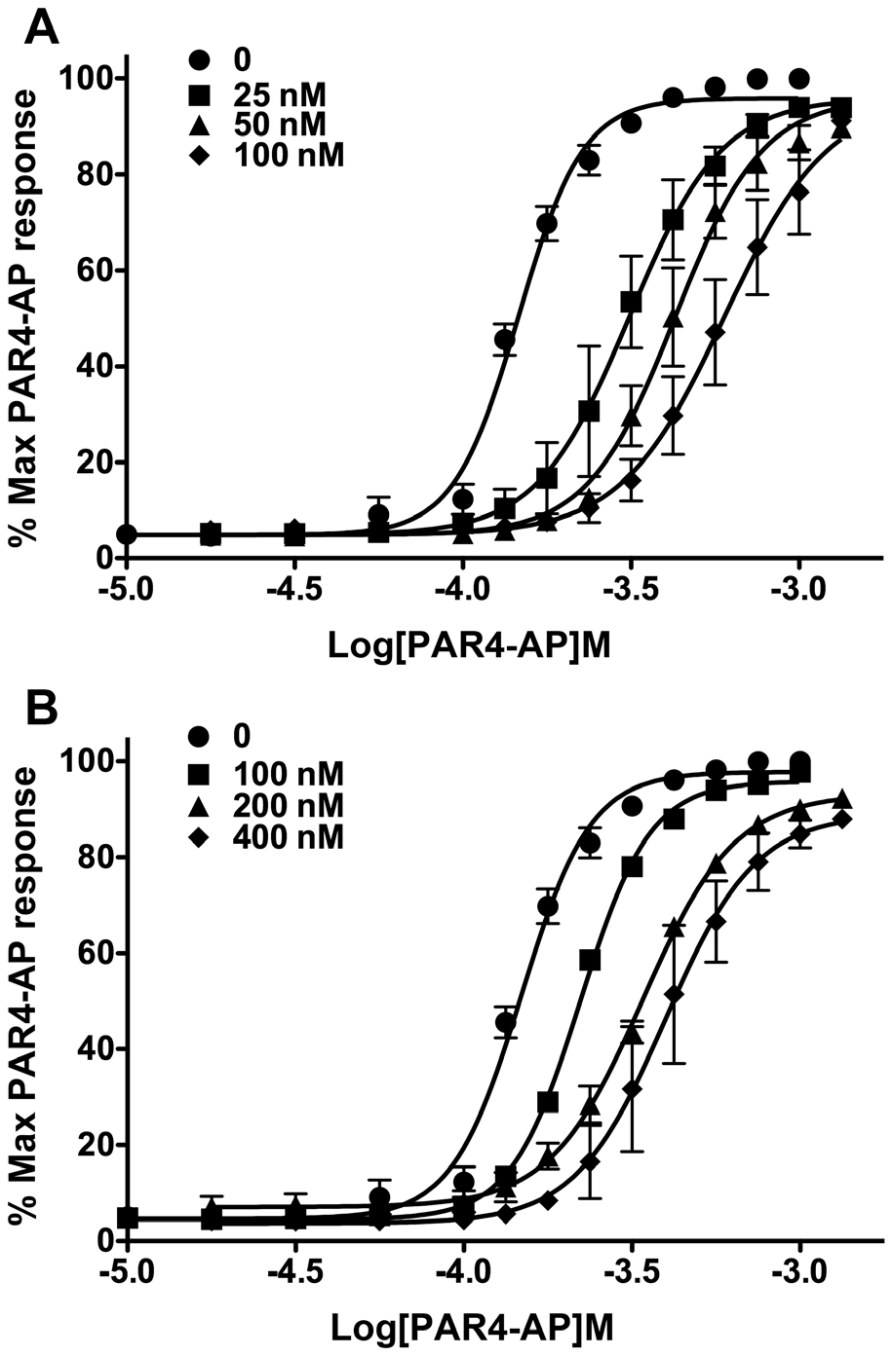
YD-3 and 1 act as competitive antagonists against PAR4. Platelets were treated with indicated concentrations of A) YD-3 or B) 1 for 5 minutes prior to stimulation with a full PAR4-AP concentration response curve. P-selectin was measured via flow cytometry. Data represented as mean±S.E.M n of 3.

To examine the putative mechanism of action of the most potent antagonists, YD-3 and 1, we used PAR4-AP mediated P-selectin expression to generate highly descriptive twelve point CRCs in the presence of increasing concentrations of antagonist. YD-3 is previously described as competitive in nature by platelet aggregation [33]. We confirmed this finding by P-selectin expression. Figure 7 shows parallel rightward shifts in the PAR4 CRC in response to increasing concentrations of compound, indicative of a competitive mechanism of action for both YD-3 (Figure 7A) and 1 (Figure 7B).

## Discussion

These data indicate that indole is a suitable scaffold for selective inhibitory activity against PAR4 mediated platelet activation and thus serve as a novel scaffold with which to build future PAR4 antagonists. Given the large number of commercially available derivatives of indole, bromides, aryl boronic acids and boronic esters, the synthetic routes presented are capable of rapidly generating numerous compounds. The use of a high throughput purified platelet calcium mobilization assay allows for rapid identification of molecules with inhibitory activity towards PAR4. Compound 1 retains antagonist activity against both the PAR4 peptide as well as thrombin and as such is the most potent novel compound presented here. The other scaffolds 3 (2-pyridine) and 5 (7-azaindole), retained selectivity for PAR4 versus PAR1 but sacrificed potency against PAR4 activating peptide and most importantly thrombin. Both YD-3 and, to a lesser extent, 1 display weak off-target effects against PAR1. The inability of compounds 1, 3, and 5 to fully inhibit PAR4 mediated platelet aggregation is reflective not of partial inhibition of the response but rather that a couple individuals fully aggregated (though slowly) in the presence of 1, 3, or 5. This phenomenon is likely reflective of the loss of affinity of these compounds for PAR4. The apparent discrepancy of YD-3 selectivity for PAR4 versus PAR1 between platelet aggregation assays and flow cytometric analysis (GPIIbIIIa activation) can be at least partially explained by signaling convergence. PAR mediated aggregation is dependent on GPIIbIIIa activation; however, ADP signaling, calcium mobilization, and granule secretion also play a significant role [17]. Additionally, there is evidence that thrombin mediated platelet aggregation, particularly PAR1-AP stimulated platelet aggregation, is resistant to GPIIbIIIa blockade [34], [35]. Gawaz et al. demonstrate using direct GPIIbIIIa blockade that approximately 50% of total GPIIbIIIa receptor is sufficient for TRAP (PAR1-AP) to elicit 80% of maximum TRAP mediated aggregation. Thus, inhibition of 55% of PAR1-AP mediated GPIIbIIIa activation may not be enough to inhibit PAR1 mediated platelet aggregation [34].

Thrombin mediated GPIIbIIIa activation was not abolished even when both antagonists were added prior to stimulation. One possible explanation is that thrombin binds GPIb receptors, which may contribute to signaling [36]. Of note, other than YD-3, PAR4 antagonists did not have a large effect against thrombin when putative PAR1 off target effects were accounted for. A probable rationale for this observation is that thrombin activated protease activated receptors create tethered ligands which are essentially irreversible agonists such that any orthosteric molecule must be either irreversible, or have such high affinity that the displacement of the tethered ligand is possible. Alternatively, one could envision that negative allosteric modulators may prove to be a better approach to inhibit thrombin activated PARs. Though we have identified indole as a favorable scaffold for future production of PAR4 antagonists, there is considerable room for improvement for antagonists of PAR4 and as such, efforts are underway to discover higher potency compounds.

## Supporting Information

Figure S1 **Names and structures of compounds synthesized.**
(PDF)

Figure S2 **Several compounds act as partial antagonists of PAR4.** Platelets were treated with indicated concentrations of compound for 5 minutes prior to stimulation with 200 µM PAR4-AP. GPIIbIIIa activation was measured via flow cytometric analysis of PAC1 binding. Hits from the second round (A) and third round (B) of optimization are shown. Data is presented as mean±S.D. n of 2 volunteers.
(TIF)

Figure S3 **Compounds display varying degrees of PAR1 off target inhibitory activity.** Platelets were treated with 10 µM of indicated antagonist for 5 minutes followed by challenge with 20 µM PAR1-AP. Flow cytometric analysis of PAC1 binding was used to determine GPIIbIIIa activation. Data presented is mean±S.D. n of 2 volunteers.
(TIF)

Figure S4 **YD-3 inhibits PAR4 mediated GPIIbIIIa activation and p-selectin expression.** Platelets were treated with YD-3 for 5 minutes prior to stimulation with 200 µM PAR4-AP. Data represented as raw mean fluorescent intensity values for platelets positive for FITC (A) or PE (B) staining. Mean±S.E.M. n of 3 volunteers is graphically represented. Calculated IC_50_ values for PAC1 binding and p-selectin expression are 17.6±1.1 nM and 16.4±1.1 nM respectively.
(TIF)
