# Supplementary material for: Synthesis of Indole Derived Protease-Activated Receptor 4 Antagonists and Characterization in Human Platelets
Source: PLoS One. 2013 Jun 11;8(6):e65528. doi: 10.1371/journal.pone.0065528 (PMC3679140; doi:10.1371/journal.pone.0065528)
Supplement: Figure S1 — Names and structures of compounds synthesized. (PDF) [file pone.0065528.s001.pdf]

Compound	Structure	Compound	Structure
YD-3	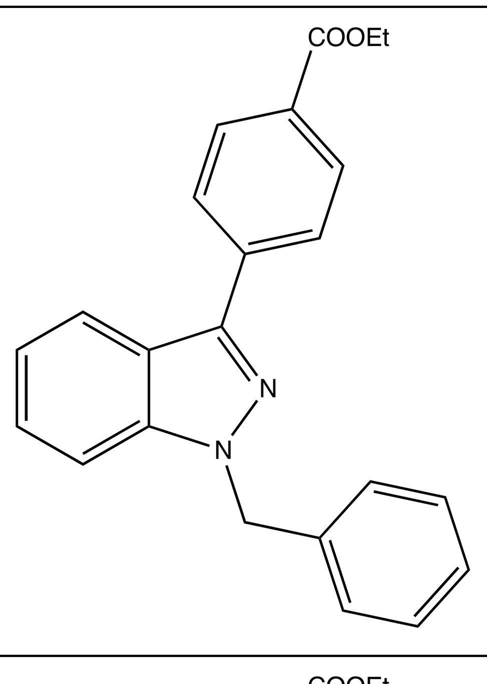	VU0469901	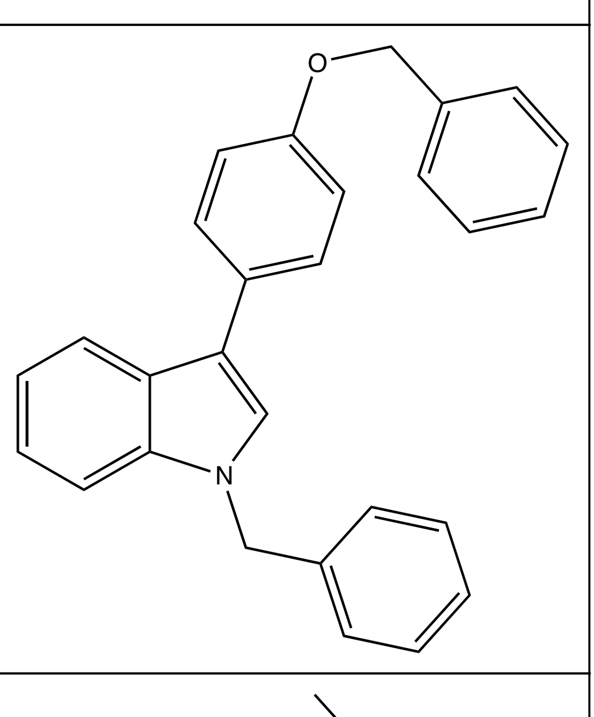
1	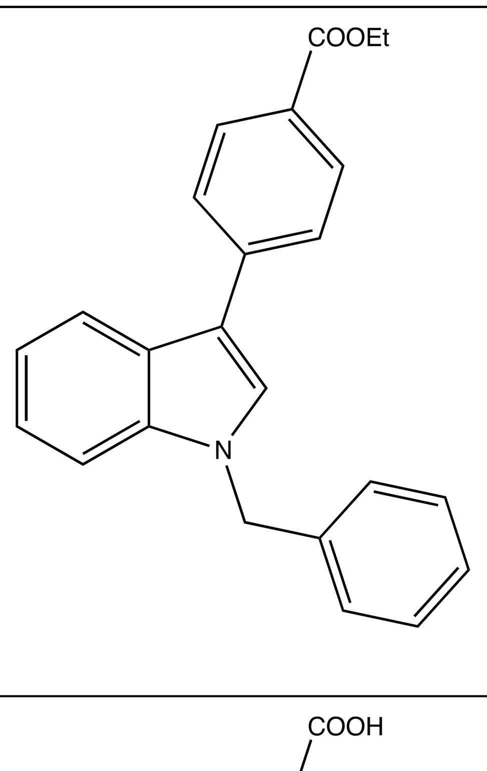	VU0469902	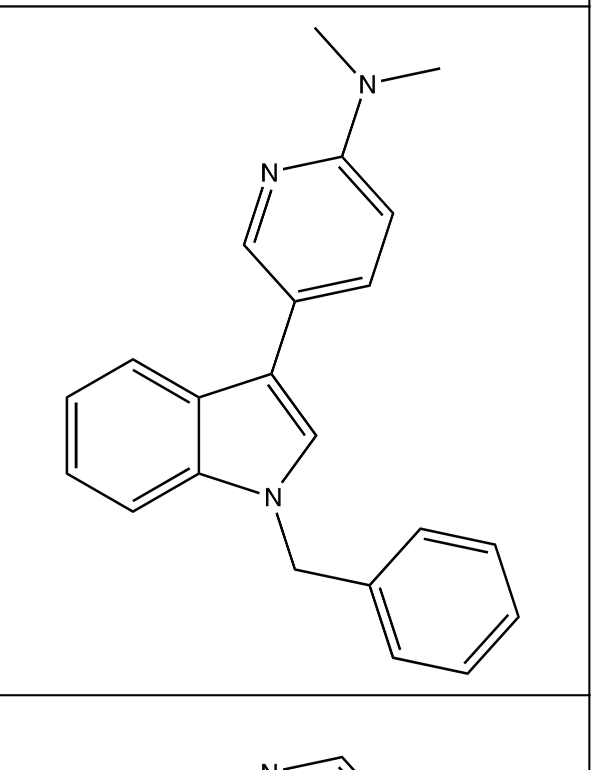
2	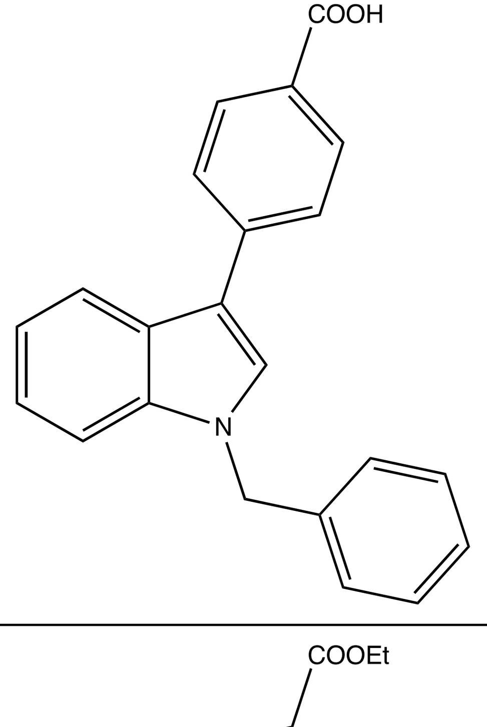	VU0469903	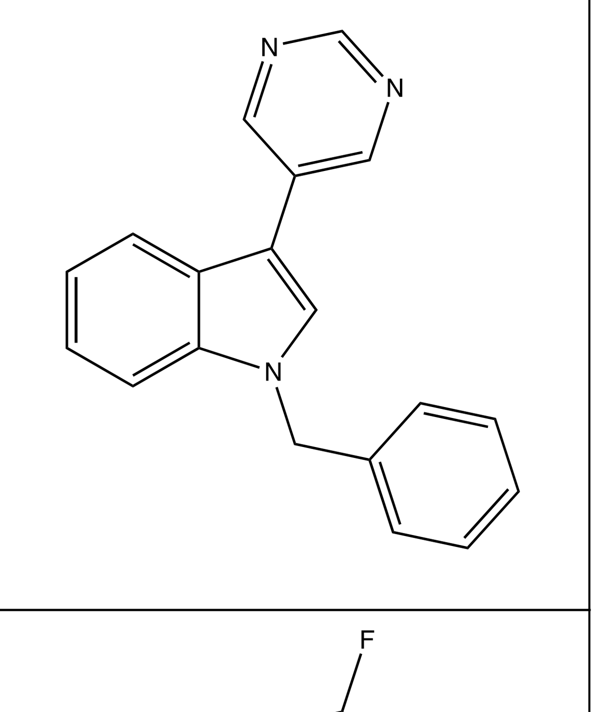
3	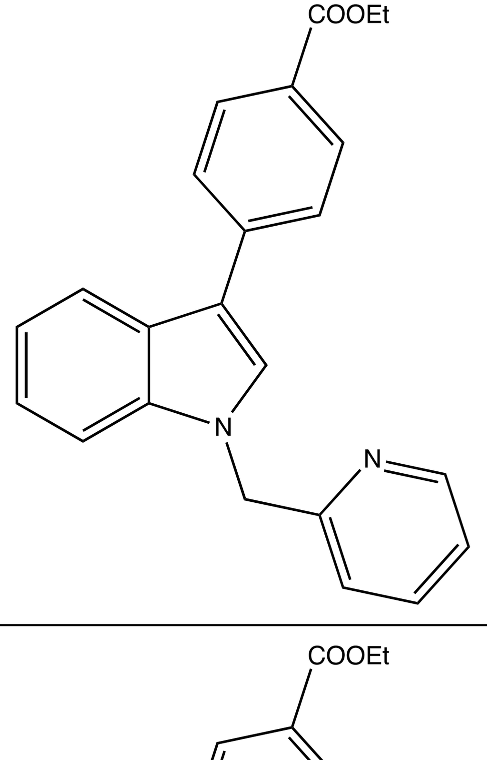	VU0469904	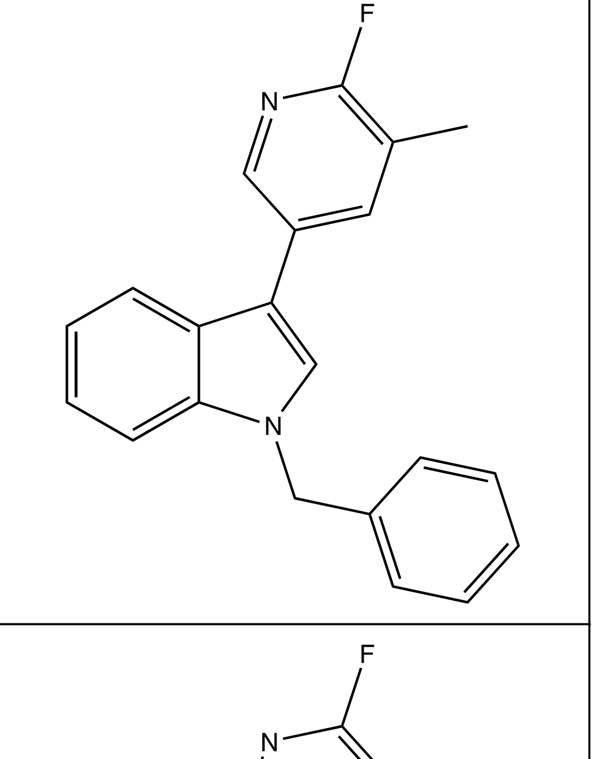
4	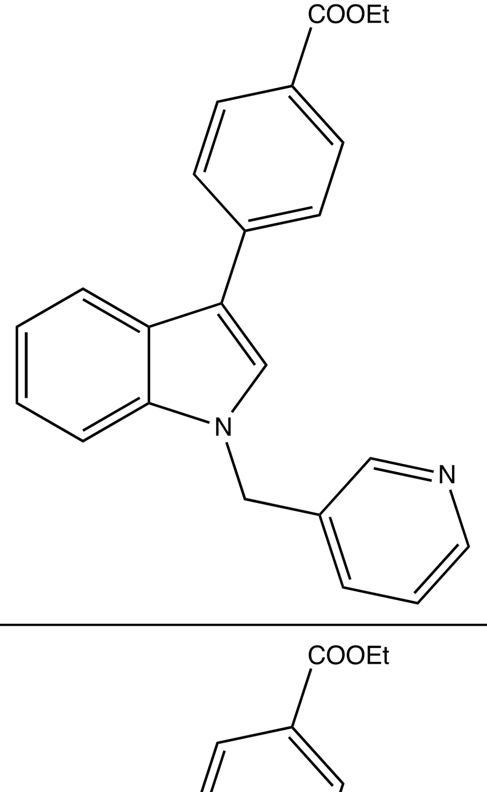	VU0469905	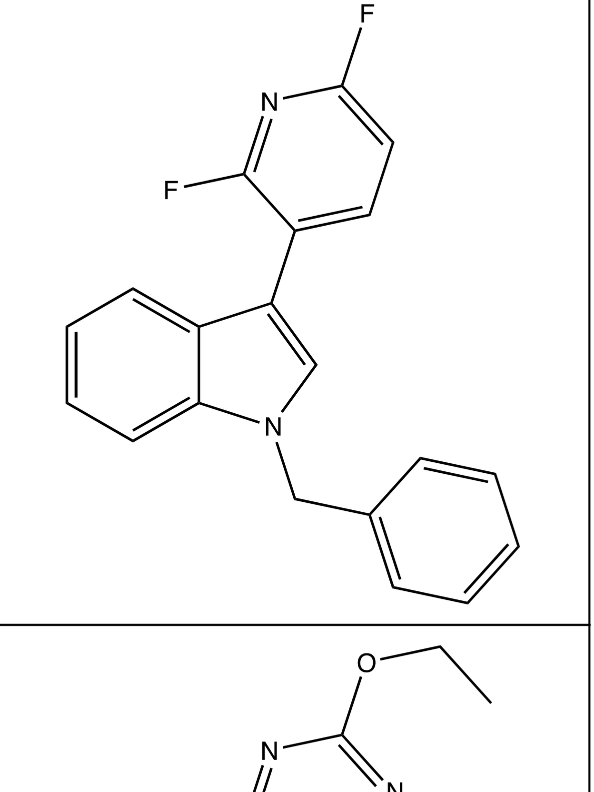
5	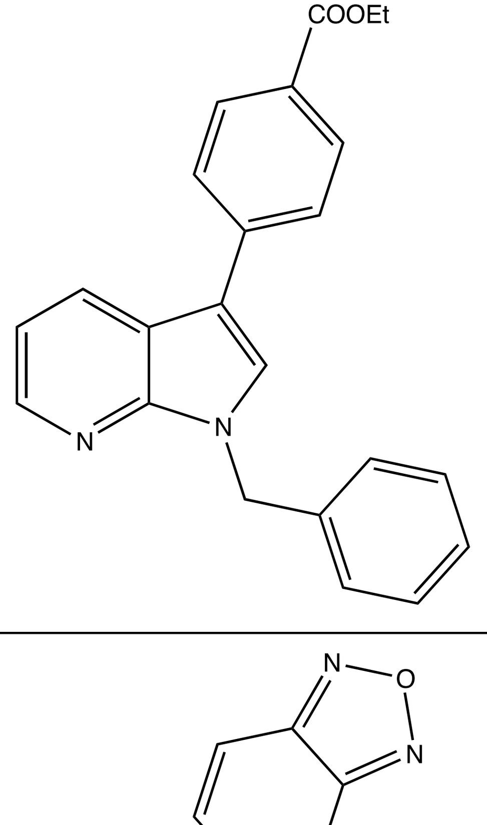	VU0469906	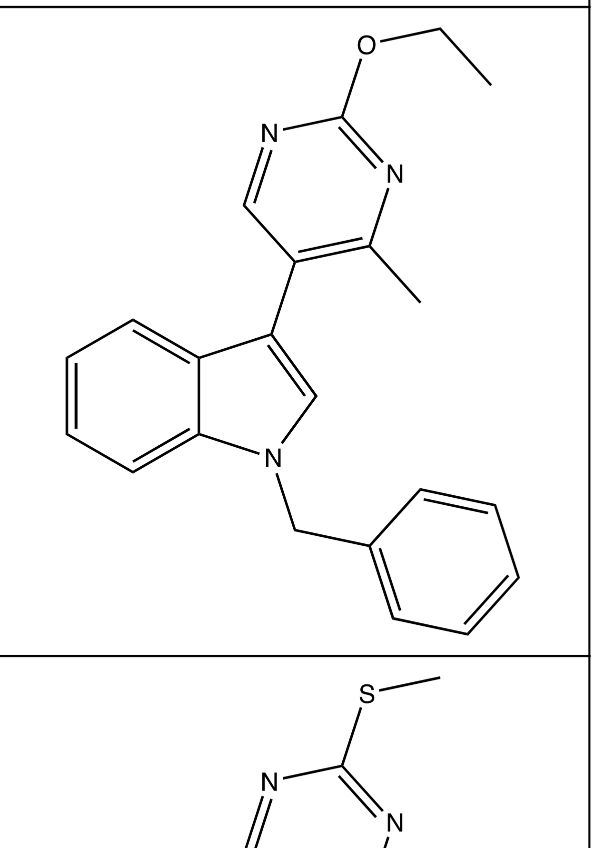
6	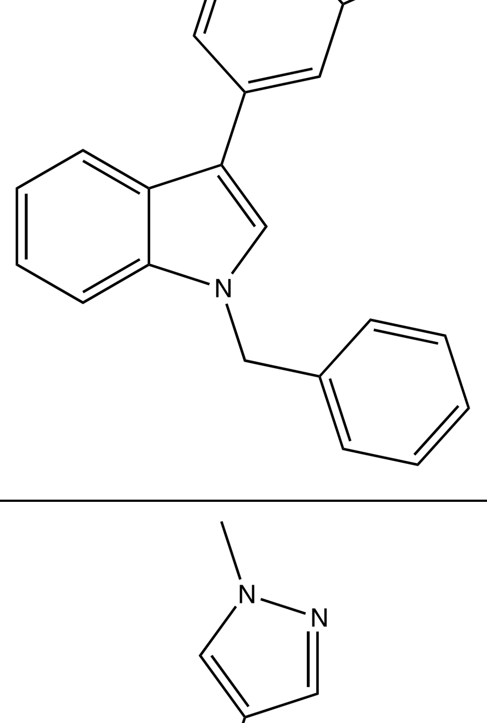	VU0469907	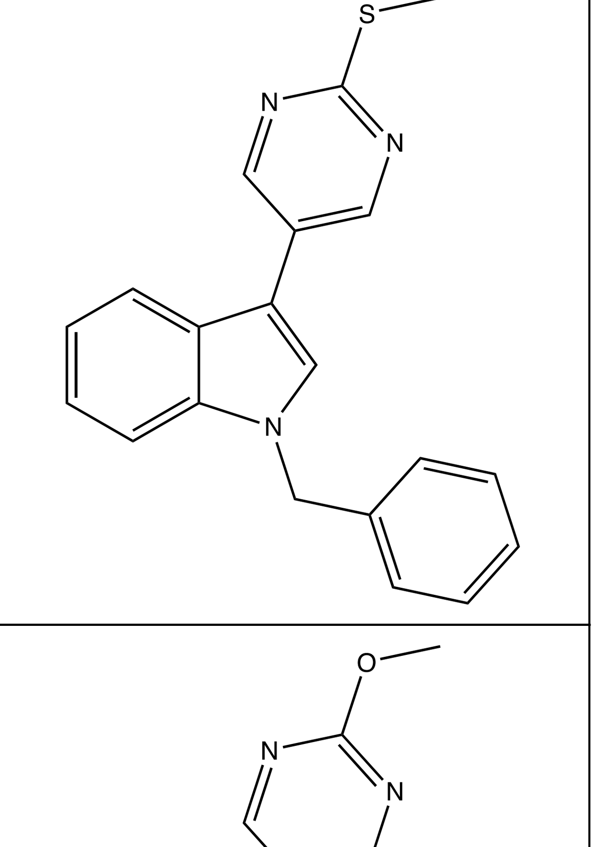
VU0469133	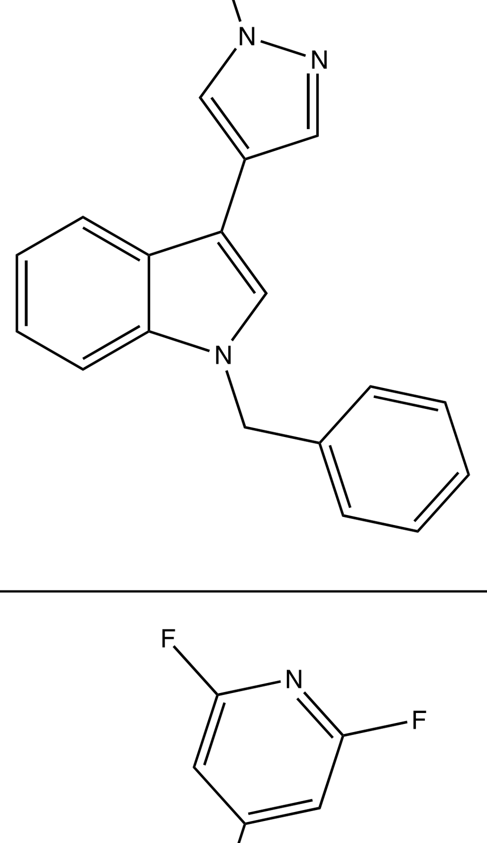	VU0469908	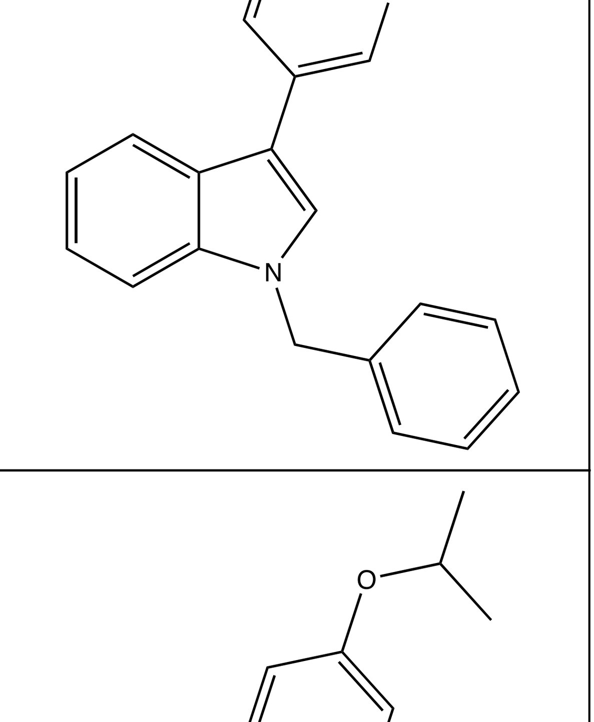
VU0469150	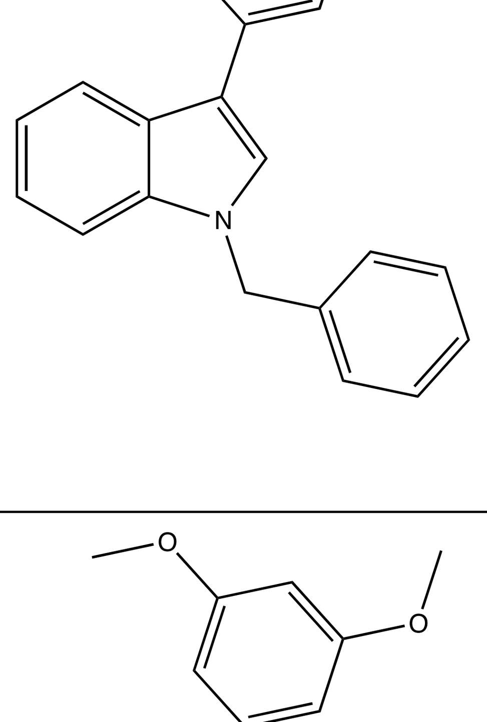	VU0469909	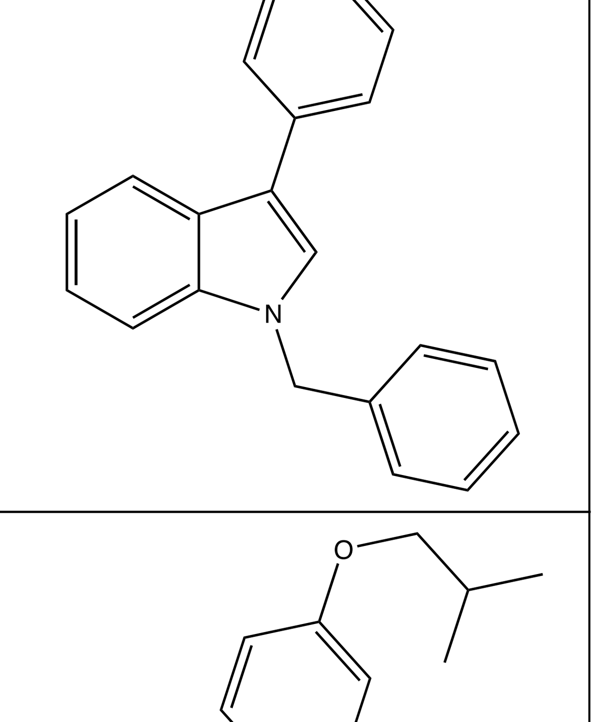
VU0469151	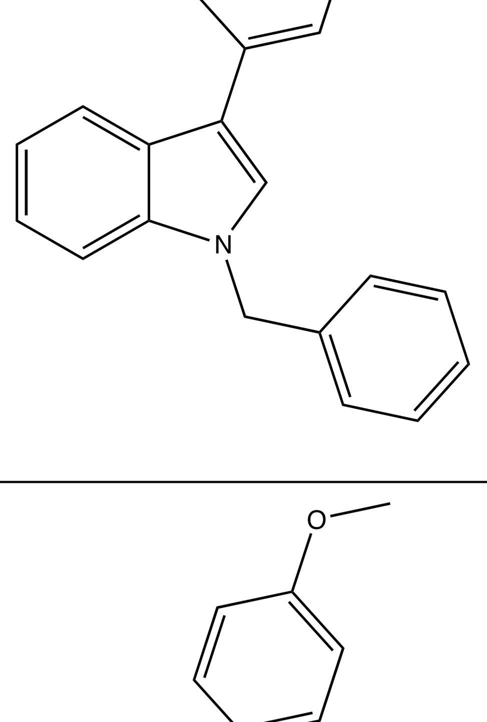	VU0469910	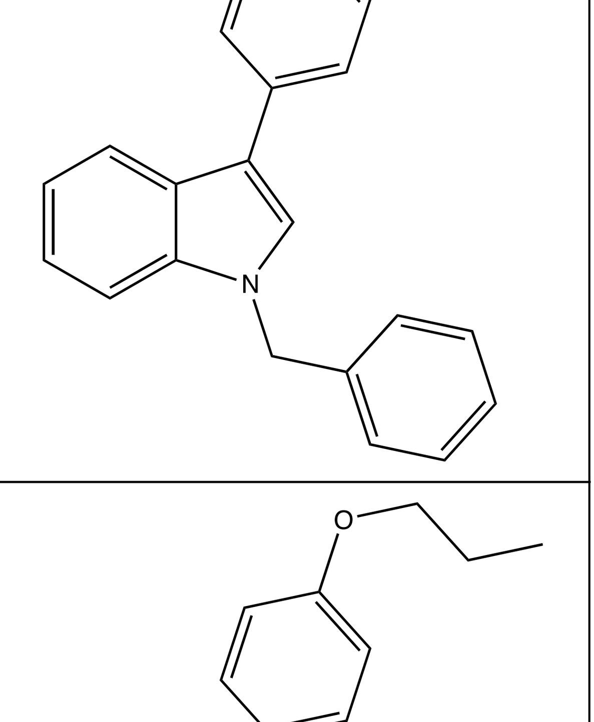
VU0469152	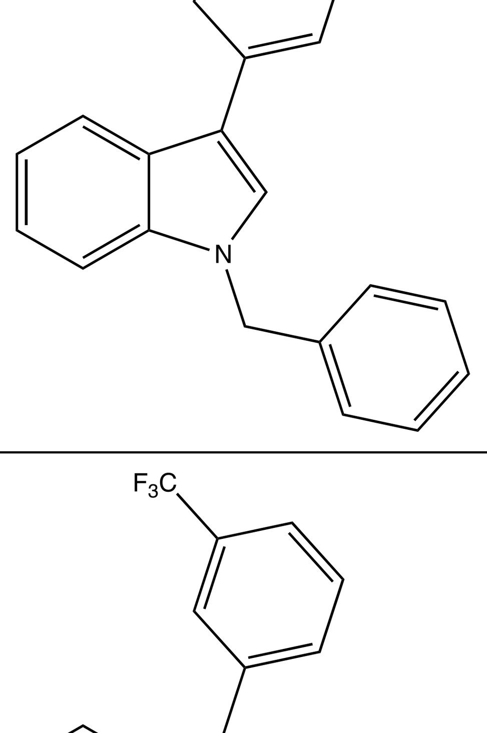	VU0469911	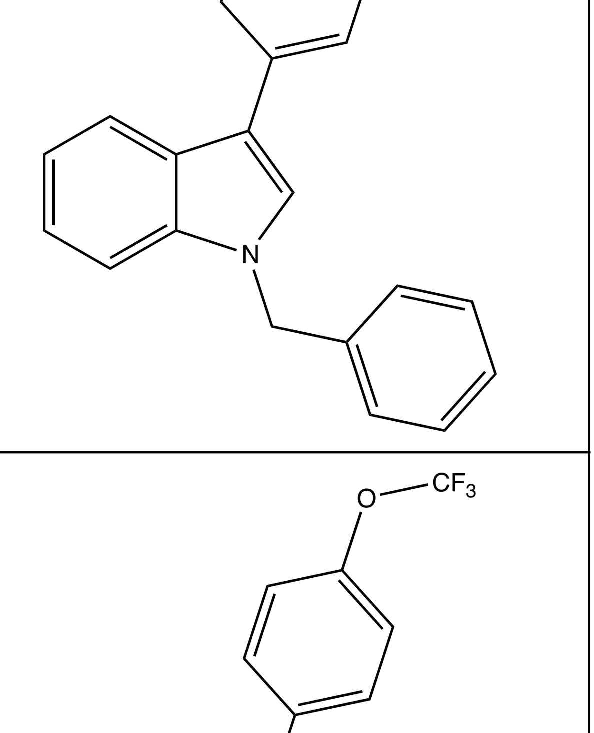
VU0469153	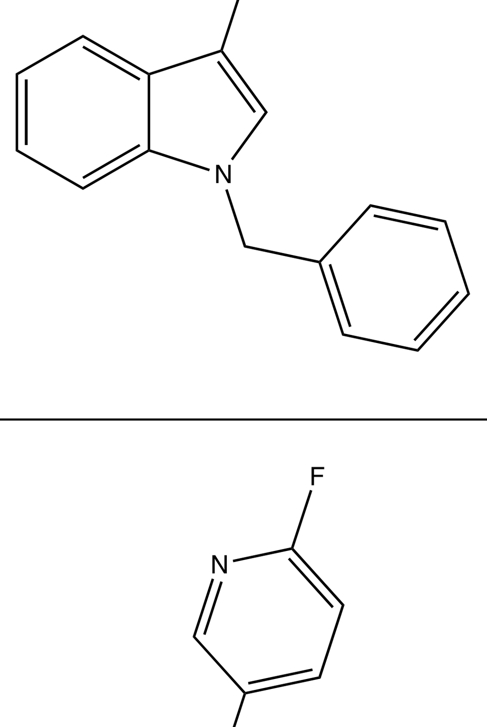	VU0469912	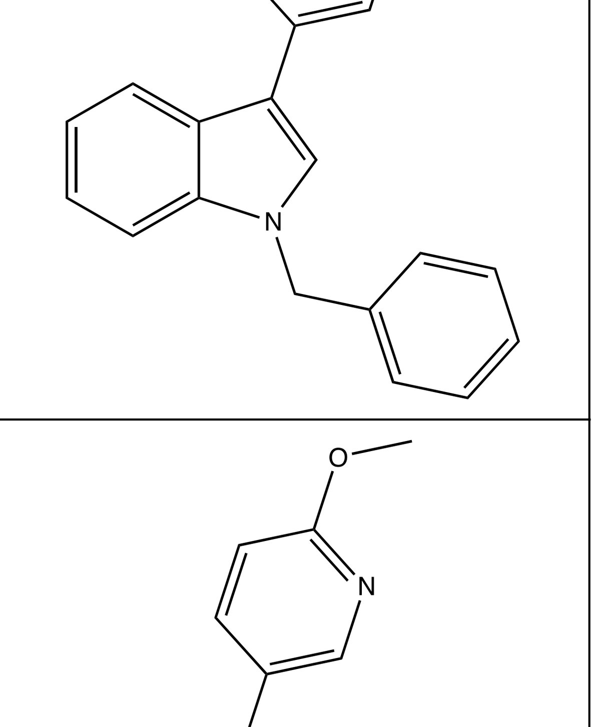
VU0469154	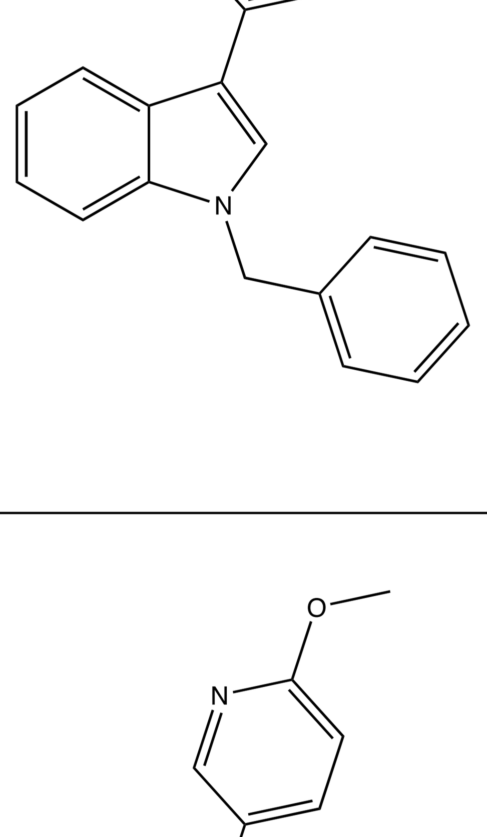	VU0476680	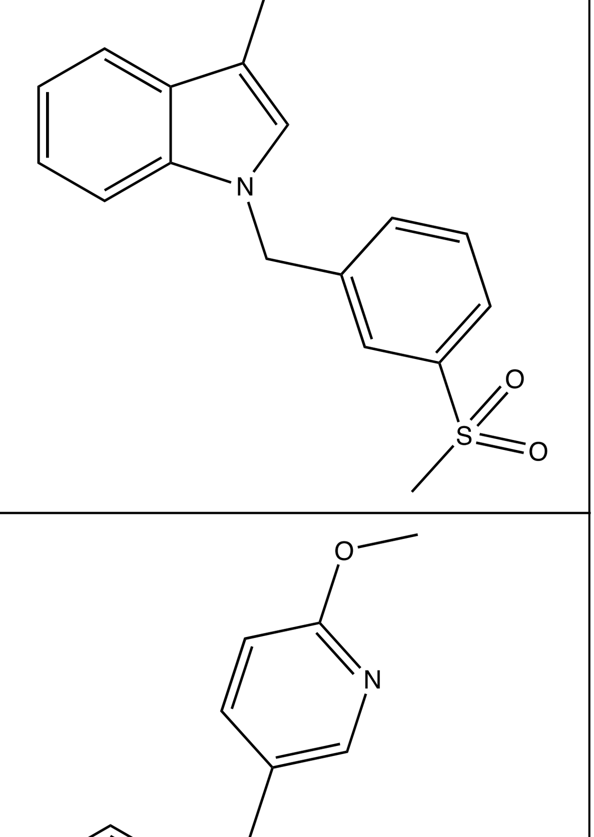
VU0469155	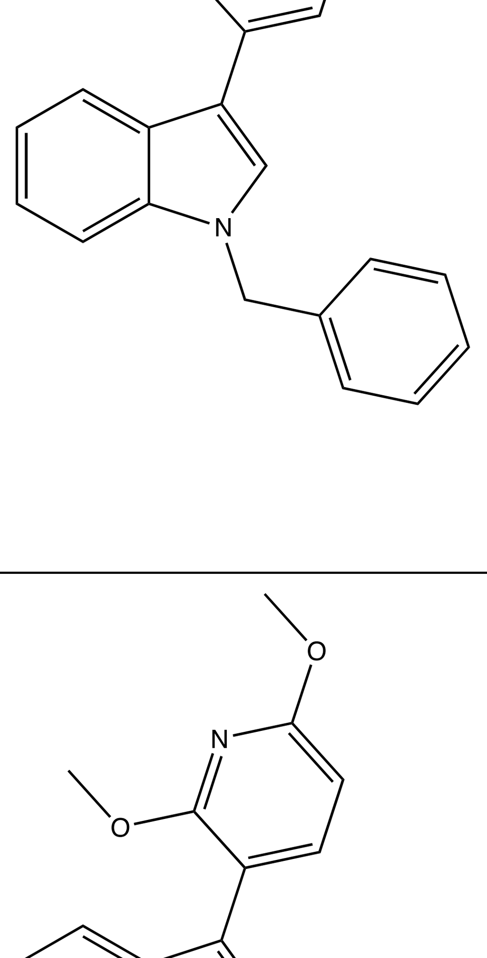	VU0476682	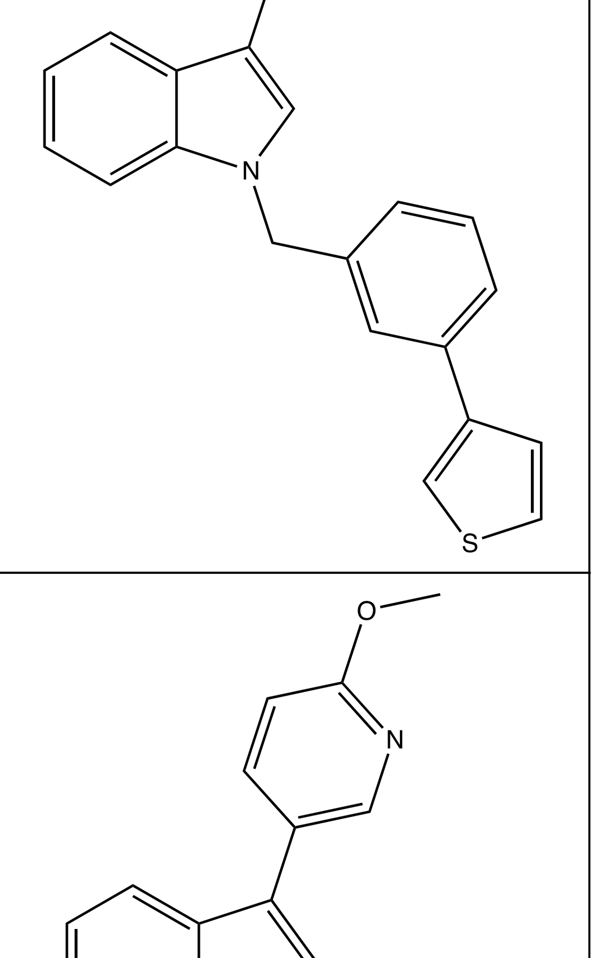
VU0469873	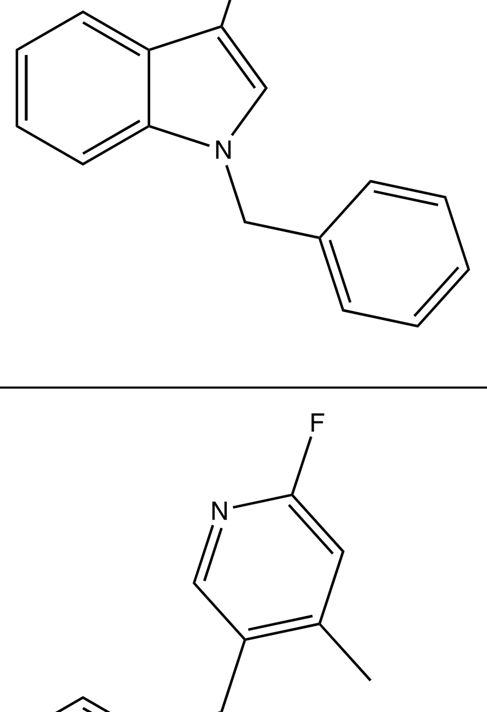	VU0476683	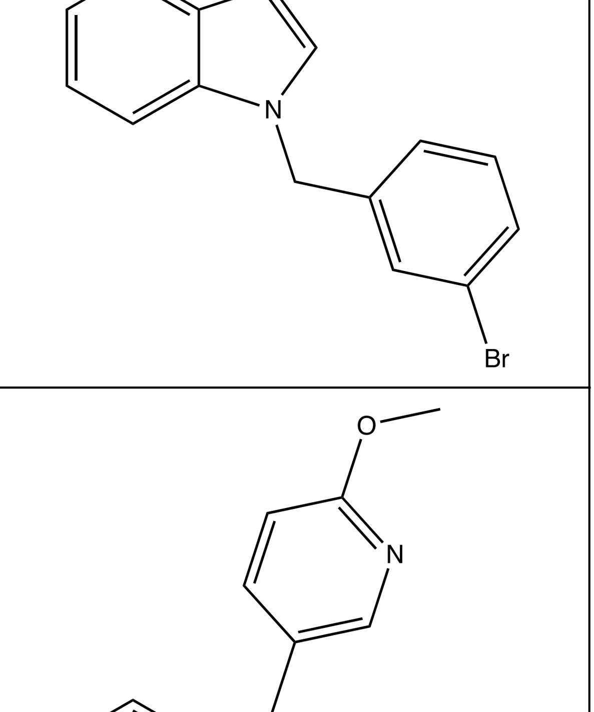
VU0469897	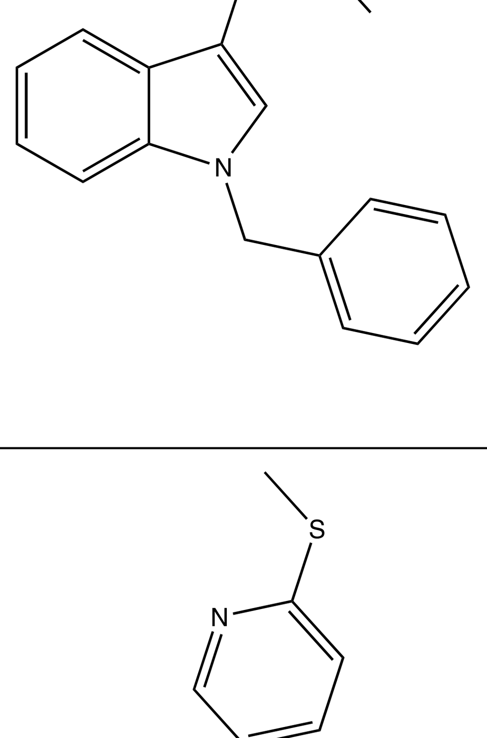	VU0476684	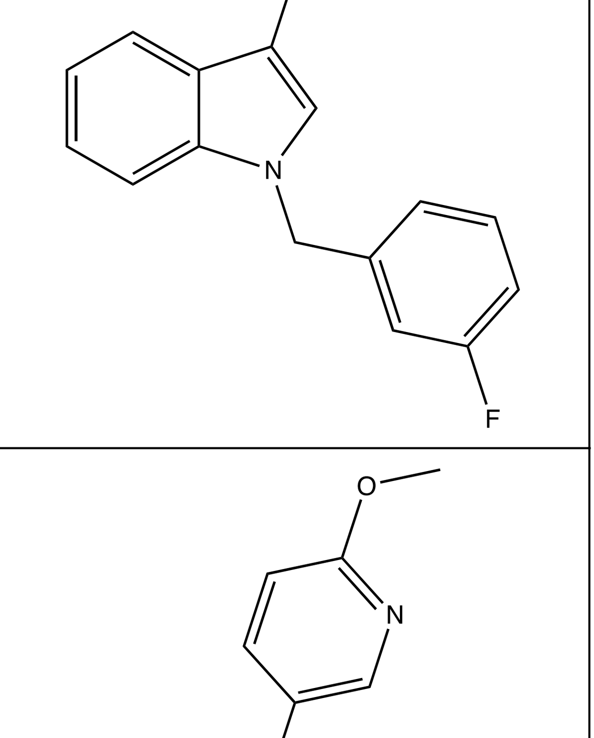
VU0469898	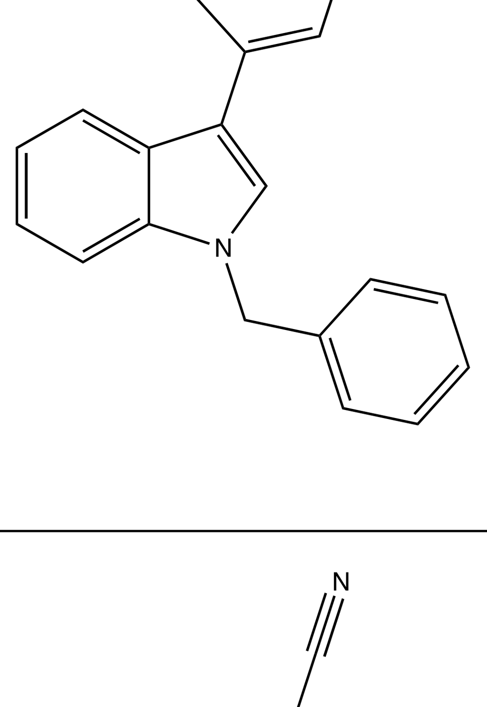	VU0476686	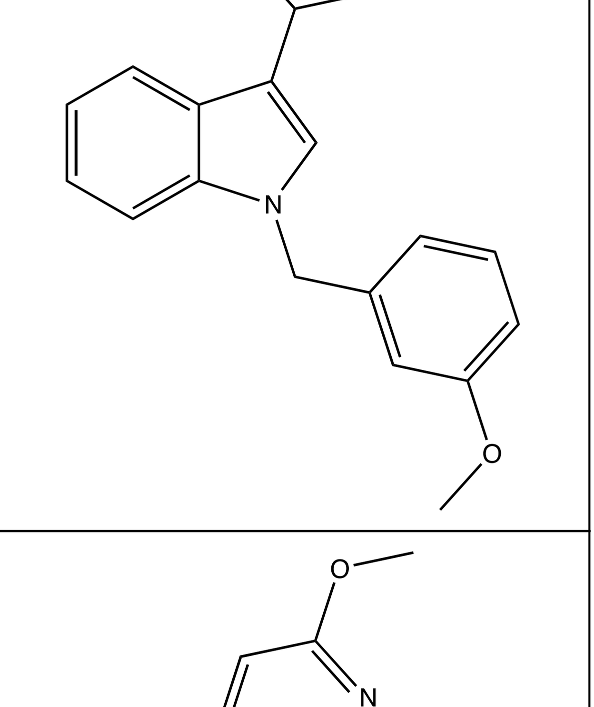
VU0469899	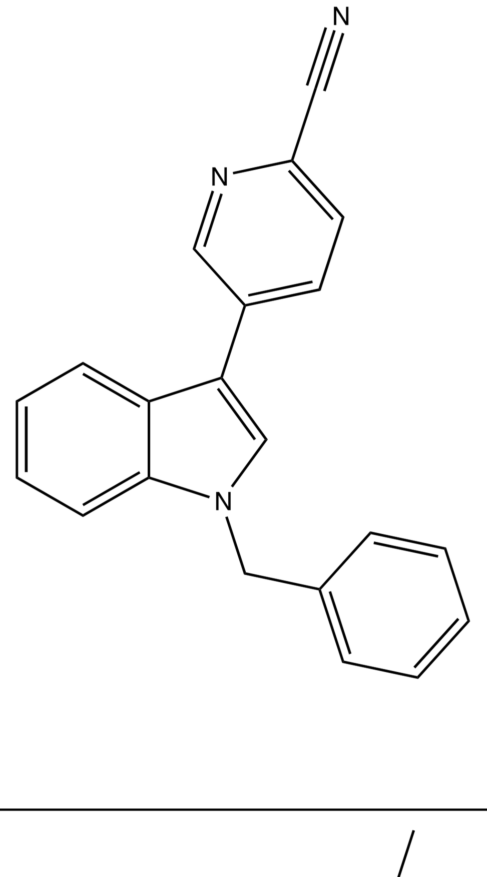	VU0476688	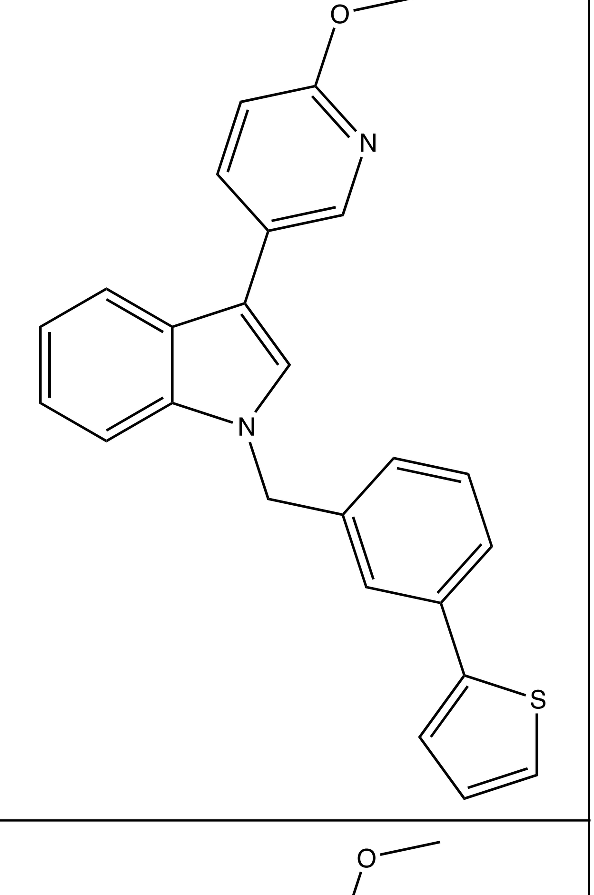
VU0469900	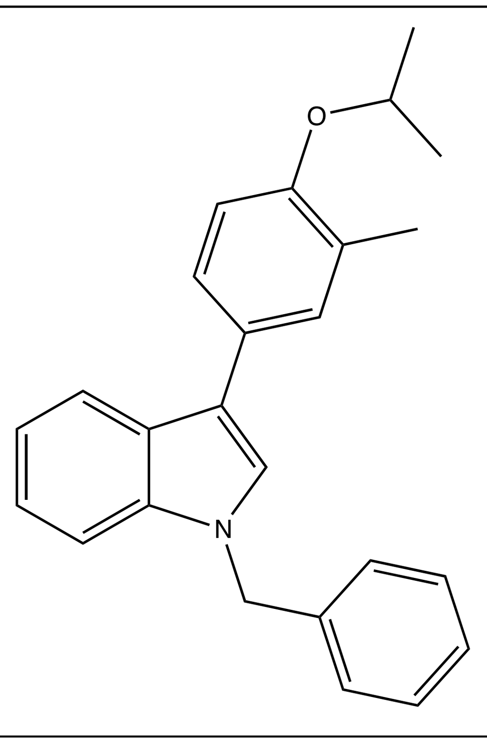	VU0476689	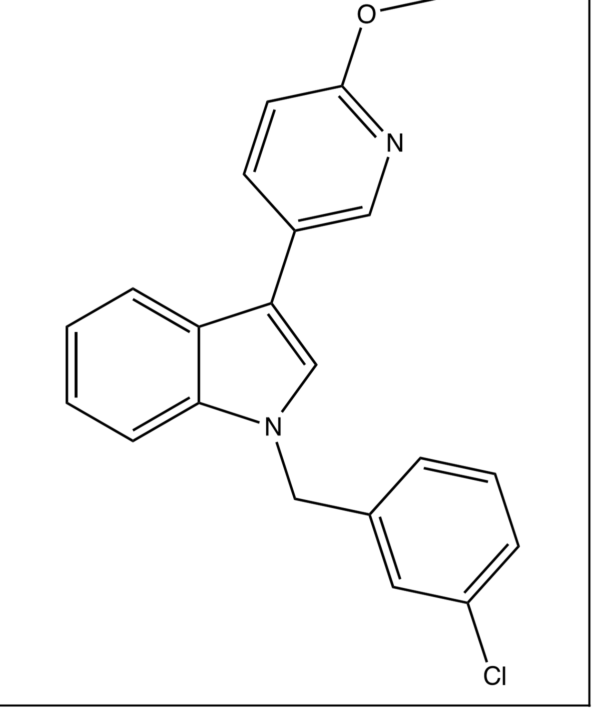
