# Supplementary figures and images for: Synthesis of Indole Derived Protease-Activated Receptor 4 Antagonists and Characterization in Human Platelets
Source: PLoS One. 2013 Jun 11;8(6):e65528. doi: 10.1371/journal.pone.0065528 (PMC3679140; doi:10.1371/journal.pone.0065528)

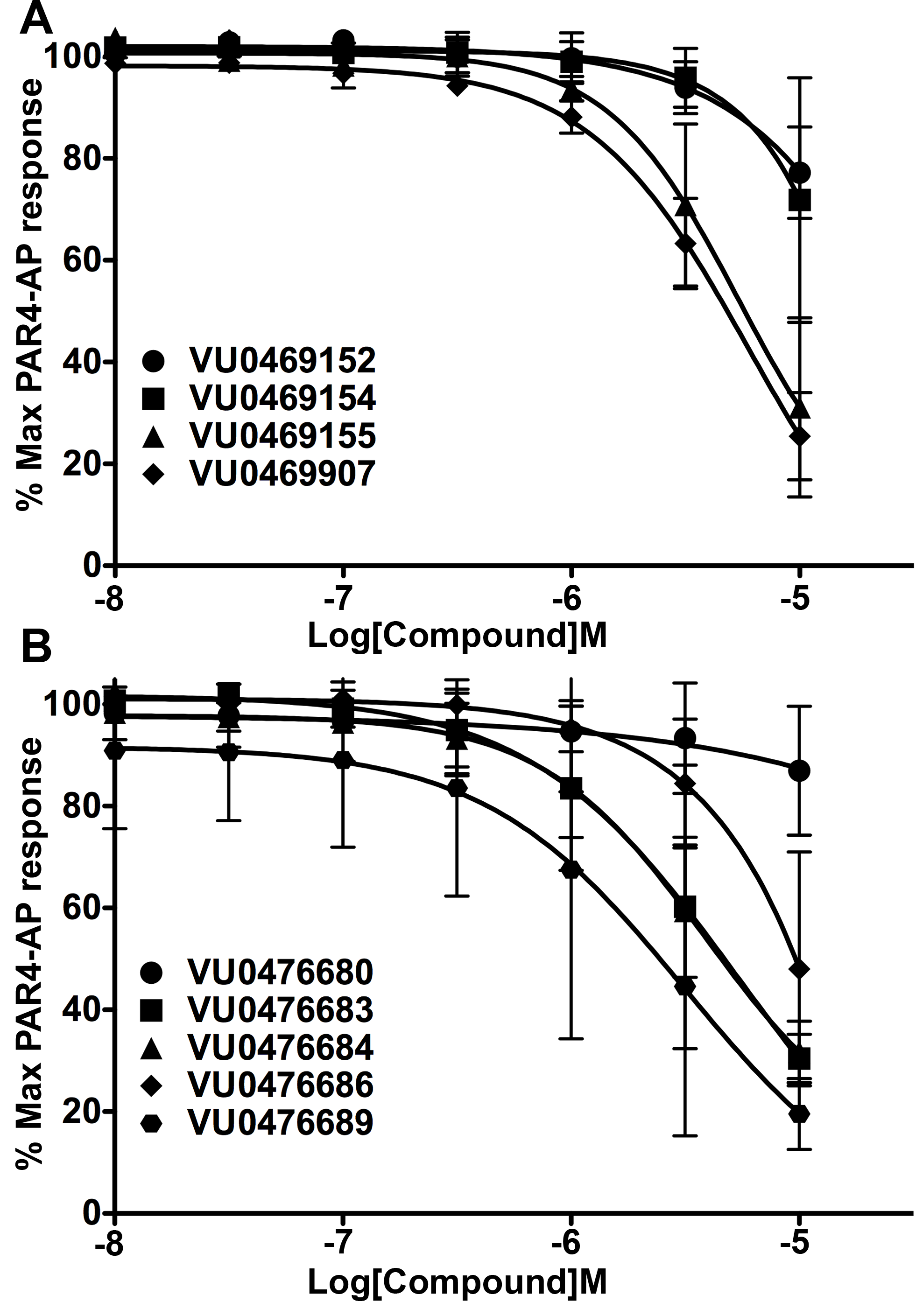

Supplement: Figure S2 — Several compounds act as partial antagonists of PAR4. Platelets were treated with indicated concentrations of compound for 5 minutes prior to stimulation with 200 µM PAR4-AP. GPIIbIIIa activation was measured via flow cytometric analysis of PAC1 binding. Hits from the second round (A) and third round (B) of optimization are shown. Data is presented as mean±S.D. n of 2 volunteers. (TIF) [file pone.0065528.s002.tif]

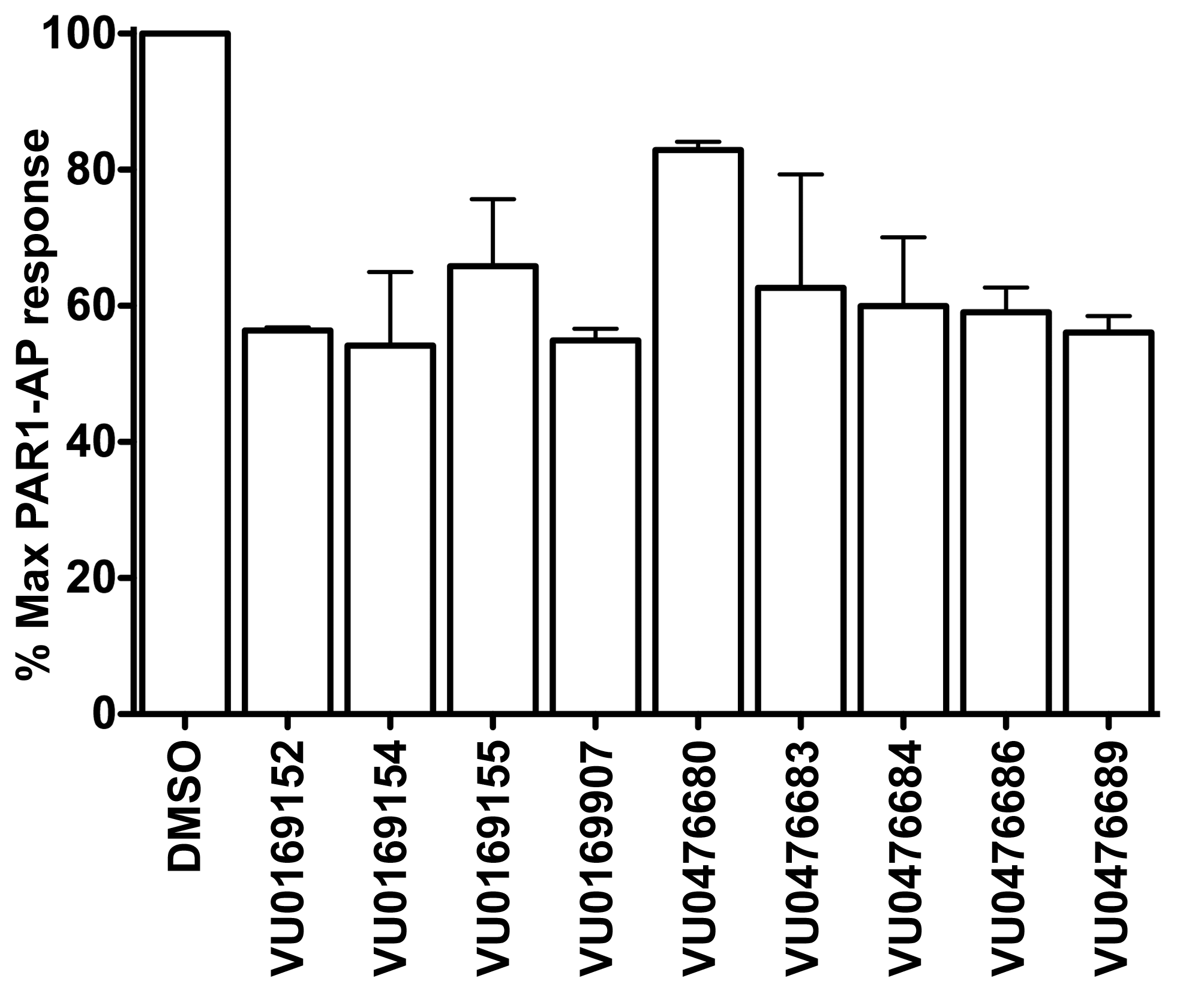

Supplement: Figure S3 — Compounds display varying degrees of PAR1 off target inhibitory activity. Platelets were treated with 10 µM of indicated antagonist for 5 minutes followed by challenge with 20 µM PAR1-AP. Flow cytometric analysis of PAC1 binding was used to determine GPIIbIIIa activation. Data presented is mean±S.D. n of 2 volunteers. (TIF) [file pone.0065528.s003.tif]
